# Oseltamivir Prophylaxis Reduces Inflammation and Facilitates Establishment of Cross-Strain Protective T Cell Memory to Influenza Viruses

**DOI:** 10.1371/journal.pone.0129768

**Published:** 2015-06-18

**Authors:** Nicola L. Bird, Matthew R. Olson, Aeron C. Hurt, Christine M. Oshansky, Ding Yuan Oh, Patrick C. Reading, Brendon Y. Chua, Yilun Sun, Li Tang, Andreas Handel, David C. Jackson, Stephen J. Turner, Paul G. Thomas, Katherine Kedzierska

**Affiliations:** 1 Department of Microbiology and Immunology, University of Melbourne, at the Peter Doherty Institute for Infection and Immunity, Parkville VIC 3010, Australia; 2 WHO Collaborating Centre for Reference and Research on Influenza, Victorian Infectious Diseases Reference Laboratory (VIDRL), at the Peter Doherty Institute for Infection and Immunity, Parkville VIC 3010, Australia; 3 Melbourne School of Population and Global Health, University of Melbourne, Parkville, Victoria 3010, Australia; 4 Department of Immunology, St Jude Children’s Research Hospital, Memphis, TN 38105, United States of America; 5 Department of Biostatistics, St Jude Children’s Research Hospital, Memphis, TN 38105, United States of America; 6 Department of Epidemiology and Biostatistics, University of Georgia, Athens, GA 30602, United States of America; 7 Federation University, School of Applied Sciences and Biomedical Sciences, Gippsland Victoria 3842, Australia; The University of Chicago, UNITED STATES

## Abstract

CD8^+^ T cells directed against conserved viral regions elicit broad immunity against distinct influenza viruses, promote rapid virus elimination and enhanced host recovery. The influenza neuraminidase inhibitor, oseltamivir, is prescribed for therapy and prophylaxis, although it remains unclear how the drug impacts disease severity and establishment of effector and memory CD8^+^ T cell immunity. We dissected the effects of oseltamivir on viral replication, inflammation, acute CD8^+^ T cell responses and the establishment of immunological CD8^+^ T cell memory. In mice, ferrets and humans, the effect of osteltamivir on viral titre was relatively modest. However, prophylactic oseltamivir treatment in mice markedly reduced morbidity, innate responses, inflammation and, ultimately, the magnitude of effector CD8^+^ T cell responses. Importantly, functional memory CD8^+^ T cells established during the drug-reduced effector phase were capable of mounting robust recall responses. Moreover, influenza-specific memory CD4^+^ T cells could be also recalled after the secondary challenge, while the antibody levels were unaffected. This provides evidence that long-term memory T cells can be generated during an oseltamivir-interrupted infection. The anti-inflammatory effect of oseltamivir was verified in H1N1-infected patients. Thus, in the case of an unpredicted influenza pandemic, while prophylactic oseltamivir treatment can reduce disease severity, the capacity to generate memory CD8^+^ T cells specific for the newly emerged virus is uncompromised. This could prove especially important for any new influenza pandemic which often occurs in separate waves.

## Introduction

Influenza viruses continually mutate, and the resultant ‘drifts’ cause seasonal epidemics, resulting in 3–5 million clinical infections and up to 500,000 deaths worldwide annually [1]. In 2009, a novel H1N1 swine-origin influenza virus spread globally and was declared the first pandemic of the 21^st^ century. Although disease severity was generally mild, this was in part a result of a significantly reduced disease burden in the elderly, attributed to cross-reactive antibody responses against pre-1957 H1N1 viruses. In contrast, the fit-young and pregnant women experienced significantly higher rates of mortality, which echoed the catastrophic 1918–19 H1N1 pandemic. Similarly, there are concerns about the possible acquisition of human-to-human transmissibility of the avian-derived H5N1 [2] and H7N9 [3,4] influenza strains that have caused severe pathological outcomes in infected individuals. Given that H5N1 and H7N9 have current case-fatality rates of 60% and 30% [5], respectively, it is clear that improved pandemic preparedness is essential.

Current influenza vaccines induce strain-specific antibodies and thus provide only transient protection due to antigenic drift. Furthermore, vaccine production takes close to six months and the composition of the vaccine must be re-evaluated and re-administered annually. This timeline complicates the ability to deliver a vaccine in a timely manner when a completely novel influenza virus emerges, as was the case in 2009. Thus, anti-influenza drugs, such as the neuraminidase inhibitor oseltamivir (Tamiflu, Roche) are stockpiled as the first line of defence against a newly emerged viral strain. Furthermore, oseltamivir prophylaxis is prescribed for those in close contact with infected individuals. Oseltamivir acts by blocking the active site of the neuraminidase (NA) glycoprotein on the surface of the virus [6]. As the enzymatic activity of the viral NA is crucial for the release of newly-synthesised virions from an infected host-cell membrane, oseltamivir ultimately acts to inhibit viral budding and further spread to neighbouring cells.

In an event of an influenza infection, CD8^+^ T cells mediate viral clearance by killing virus-infected cells and through the release of antiviral cytokines such as IFN-γ, TNF-α, and IL-2 [7]. In contrast to neutralising antibodies, CD8^+^ T cells directed toward the more conserved internal viral antigens can elicit cross-strain responses to ameliorate disease severity upon re-infection with HA- and NA-distinct viruses. A role for CD8^+^ T-cells in protecting against heterologous challenge was shown between H1N1, H7N7, H3N2, H5N1 and H7N9 viruses [8–13]. Furthermore, the relative ‘mildness’ of the H1N1pdm09 was associated with the high conservation of CD8^+^ T cell epitopes between the swine-origin influenza and circulating seasonal strains [14–16].

Given the evident importance of CD8^+^ T cells in cross-strain immunity to influenza infection and the poor CD8^+^ T cell response generated by current influenza vaccines, there is a need to understand how effective CD8^+^ T cell memory to influenza viruses is generated. Our previous studies suggest that functional influenza-specific CD8^+^ T cell memory can be established early, within the first three days of a ‘natural’ course of infection [17–19]. However, it is unclear whether an uninterrupted, ‘natural’, course of influenza infection is necessary for the establishment of memory CD8^+^ T cells. Given the dependence on anti-viral treatment in the face of a pandemic, an important question is whether ‘interrupting’ an influenza infection by antiviral treatment affects the establishment of long-lived, functional memory CD8^+^ T cells. Here, we used a well-characterised C57BL/6J (B6) mouse model of influenza infection, ferrets and longitudinal human H1N1 patient samples to understand how T cell immunity to influenza virus is generated during prophylactic and ongoing oseltamivir treatment. Our data suggest that prophylactic oseltamivir treatment, although causing a marked reduction in influenza-related morbidity, inflammation, and effector CD8^+^ and CD4^+^ T cell responses, permits the generation of functional, long-lived, and cross-strain responsive memory CD8^+^ and CD4^+^ T cell pools. Of note, this improvement in morbidity and inflammation was observed even in the absence of strong effects on measured viral titre. Thus, prophylactic oseltamivir treatment reduces disease severity without compromising the capacity to generate memory T cells to the newly emerged virus.

## Results

### Prophylactic oseltamivir treatment ameliorates disease severity in influenza-infected mice

**Fig 1:**
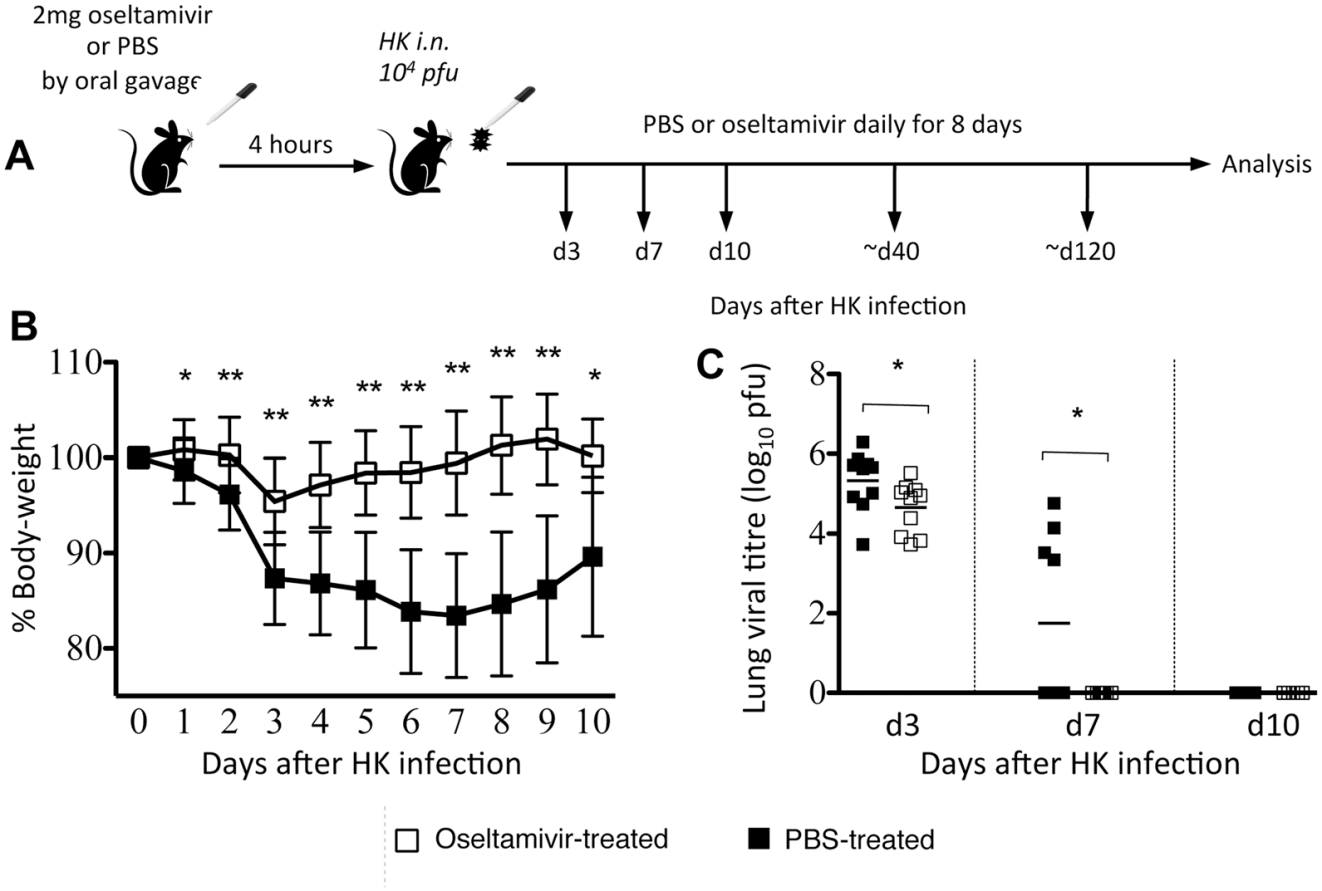
Oseltamivir prophylaxis ameliorates disease severity by reducing weight loss and accelerating viral clearance in influenza virus-infected mice. (A) Naïve female BL/6 mice were orally administered either 2mg oseltamivir in PBS or PBS alone 4 hrs prior to i.n. infection with 10^4^ pfu of HK, and once daily for 8 days thereafter. (B) Mice were monitored daily for weight loss. Data represent the mean and standard deviation of 8 separate experiments each with 4–5 mice per group. (C) Lungs were homogenised and clarified supernatants were plaqued on MDCK cell monolayers to determine viral titres. Symbols denote individual mice and the mean is shown for each group. Data were pooled from 2 independent experiments each with 4–5 mice per group. *P<0.05, **P<0.01.

To understand the effect of antiviral prophylaxis on the establishment of influenza-specific T cell memory, we first determined how oseltamivir treatment impacts influenza disease severity. B6 mice intranasally (i.n.) infected with 10^4^ pfu of HK (H3N2; HK- X31) were administered 2mg of oseltamivir or PBS orally 4-hours prior to infection, and then daily for 8 days (d) (Fig 1A). The animals were monitored daily for morbidity, as measured by a loss of body-weight. Consistent with our previous studies, the PBS-treated control mice lost ~15–20% of body-weight by d7 post-infection and showed clinical symptoms of disease such as inactivity, hunched back and ruffled fur. In contrast, the oseltamivir-treated mice showed early modest weight loss (~5% at d3 post-infection, followed by a rapid recovery) (Fig 1B) and remained fully active throughout the infection.

The antiviral effect of oseltamivir was confirmed by a plaque assay of clarified lung homogenates prepared from virus-infected mice at d3, d7 and d10 after infection (Fig 1C). Although oseltamivir-treated mice had only a modest decrease in total lung viral load at d3, these mice displayed accelerated virus clearance on d7. Conversely, only some mice in the PBS-treated group had cleared virus by d7, with all mice clearing virus at d10. Thus, in a mouse model of infection, oseltamivir-prophylaxis reduces the severity of influenza disease, modestly reduces early viral load, and accelerates viral clearance.

### Reduced morbidity in oseltamivir-treated mice correlates with markedly reduced influenza-induced inflammation at the site of infection

**Fig 2:**
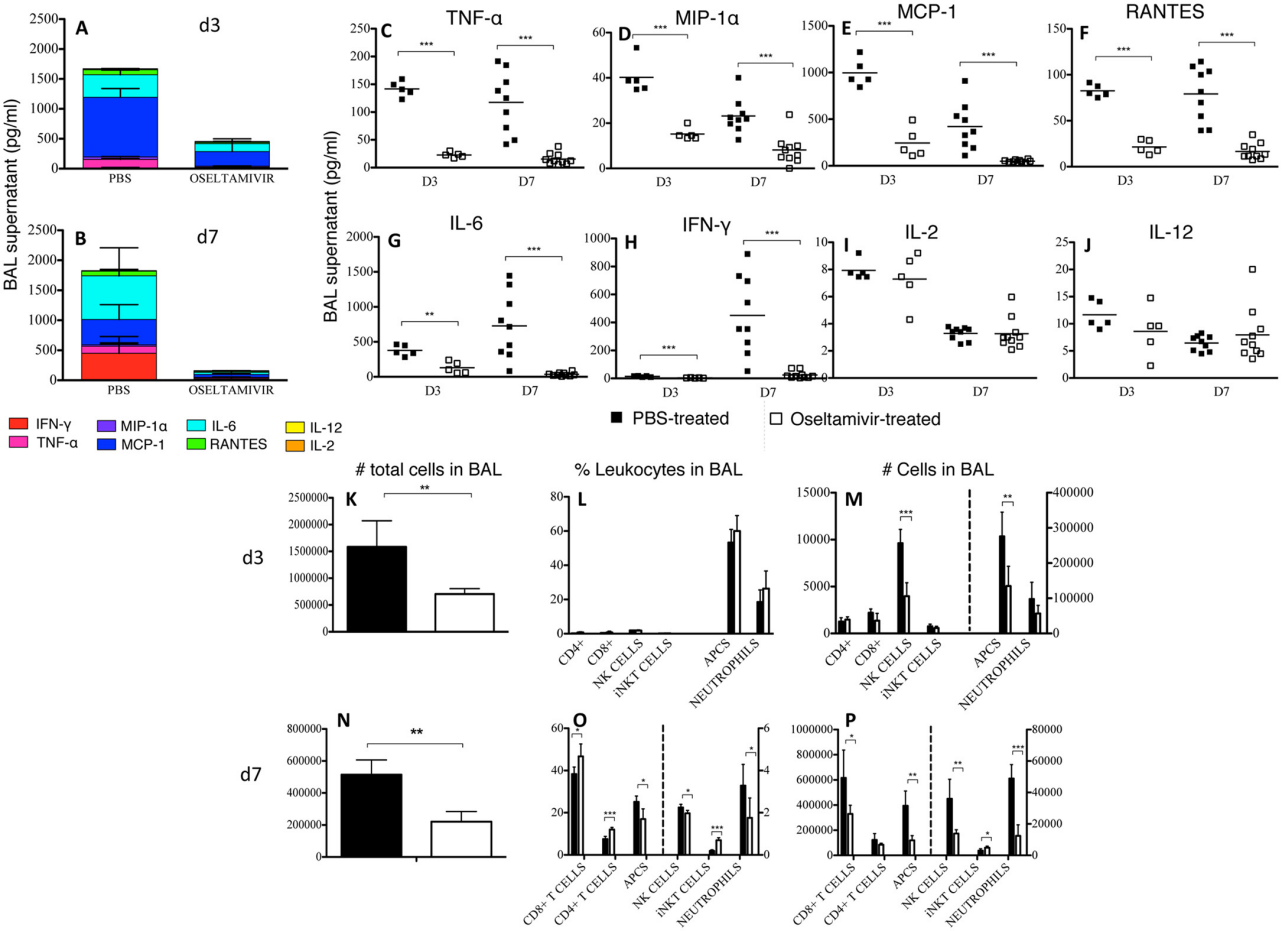
Reduced cytokine/chemokine levels and cellular recruitment to the airways of oseltamivir-treated mice. Mice received oseltamivir or PBS 4 hours prior to infection with 10^4^ pfu of HK and once daily for 8 days thereafter. Clarified BAL supernatants were assayed by cytometric bead array for cytokine/chemokine concentration. Cumulative amounts and relative contribution of each cytokine or chemokine are demonstrated at (A) day 3, or (B) day 7 post-influenza infection in PBS- or oseltamivir-treated mice. Levels of (C) TNF-α, (D) MIP-1A, (C) MCP-1, (F) RANTES, (G) IL-6, (H) IFN-γ, (I) IL-2, and (J) IL-12 in PBS- or oseltamivir-treated mice are also shown. Data comprise one to two separate experiments per time-point, each with 4–5 mice per group. Immune cells were enumerated by antibody staining and cell counts at (K-M) 3 days, and (N-P) 7 days after infection. *P<0.05; **P<0.01; ***P<0.001.

Given that the cytokine storm is a known contributor to the clinical severity of influenza disease, we assessed the cytokine/chemokine profiles of oseltamivir- and PBS-treated influenza-infected mice at d3 and d7 (Fig 2). Cumulative cytokine concentrations in clarified BAL supernatants of oseltamivir-treated mice showed significant reductions in the total amount of inflammatory cytokines/chemokines at both d3 (3-fold reduction) (Fig 2A) and d7 (6-fold reduction) (Fig 2B). The total amount of cytokine/chemokines in PBS-treated mice was similar between the two time-points after infection, whereas the amount of cytokines/chemokines in oseltamivir-treated mice decreased by approximately 50% from d3 to d7, suggesting both a diminished and less-sustained inflammatory response in these mice. Overall, the significant reduction in inflammatory TNFα (Fig 2C), MIP-1α (Fig 2D), MCP-1 (Fig 2E), RANTES (Fig 2F), IL-6 (Fig 2G) and IFN-γ (Fig 2H) in the lungs of oseltamivir-treated mice can explain, at least in part, the much milder symptoms of influenza virus infection following oseltamivir prophylaxis. Furthermore, these findings suggest that although a substantial viral load was present at d3, the virus was controlled by oseltamivir in a way that did not induce a profound inflammatory environment in the infected lung.

The decreased inflammatory milieu at the site of infection was suggestive of a diminished cellular infiltrate to the lungs of influenza-infected, oseltamivir-treated mice. To demonstrate this, we determined the proportions and total numbers of NK cells, neutrophils, iNKT cells, CD4^+^, and CD8^+^ T cells, as well as APCs by antibody staining and cell counts (Fig 2K–2P). By d3 after infection, the total leukocyte number in BAL of mice treated with oseltamivir was significantly reduced compared to the PBS-treated control mice (Fig 2K). There were no differences between oseltamivir-treated and PBS-treated mice in the frequencies of each cell-type (Fig 2L); so the reduction in the numbers for each individual cell population is proportional to the drop in the total cell count (Fig 2M). Thus, the early immune response mounted by oseltamivir-treated mice was qualitatively similar to that of PBS-treated mice, only reduced in magnitude.

At d7 after infection, both the frequency and number of APCs, NK cells and neutrophils were diminished in oseltamivir-treated mice (Fig 2N–2P). Proportionally, there were more CD8^+^ T cells in the lungs of oseltamivir-treated mice, however, this likely only reflects a greater frequency reduction for innate cells, as the total number of CD8^+^ T cells recruited to the site of influenza infection in oseltamivir-treated mice was also significantly reduced.

In sum, our data show a marked decrease in both the innate and adaptive immune response at the site of influenza infection in oseltamivir-treated mice.

### Oseltamivir treatment does not significantly reduce viral load in humans

To verify our mice findings in humans, we assessed the effects of oseltamivir treatment in a previously described human cohort (FLU09) [20]. This cohort was obtained in a case-based ascertainment household study, with index cases symptomatic for less than four days being enrolled along with any household contacts. Nasal swabs and nasal washes were obtained from all participants at enrollment. Influenza-positive individuals were re-sampled at or around days 3, 7, 10, and 28. Influenza-negative household contacts were swabbed on days 3, 7, 10, and 14, and, if a positive-result was obtained, were enrolled as a case and sampled as influenza-positive index cases. Information on concomitant medications was recorded at each visit. Patients were divided into those who reported no medication, those who reported oseltamivir usage (but not NSAIDs), and those who reported NSAID usage (but not oseltamivir). We excluded individuals who were on both types of drug and all individuals who were taking any steroid medication.

**Fig 3:**
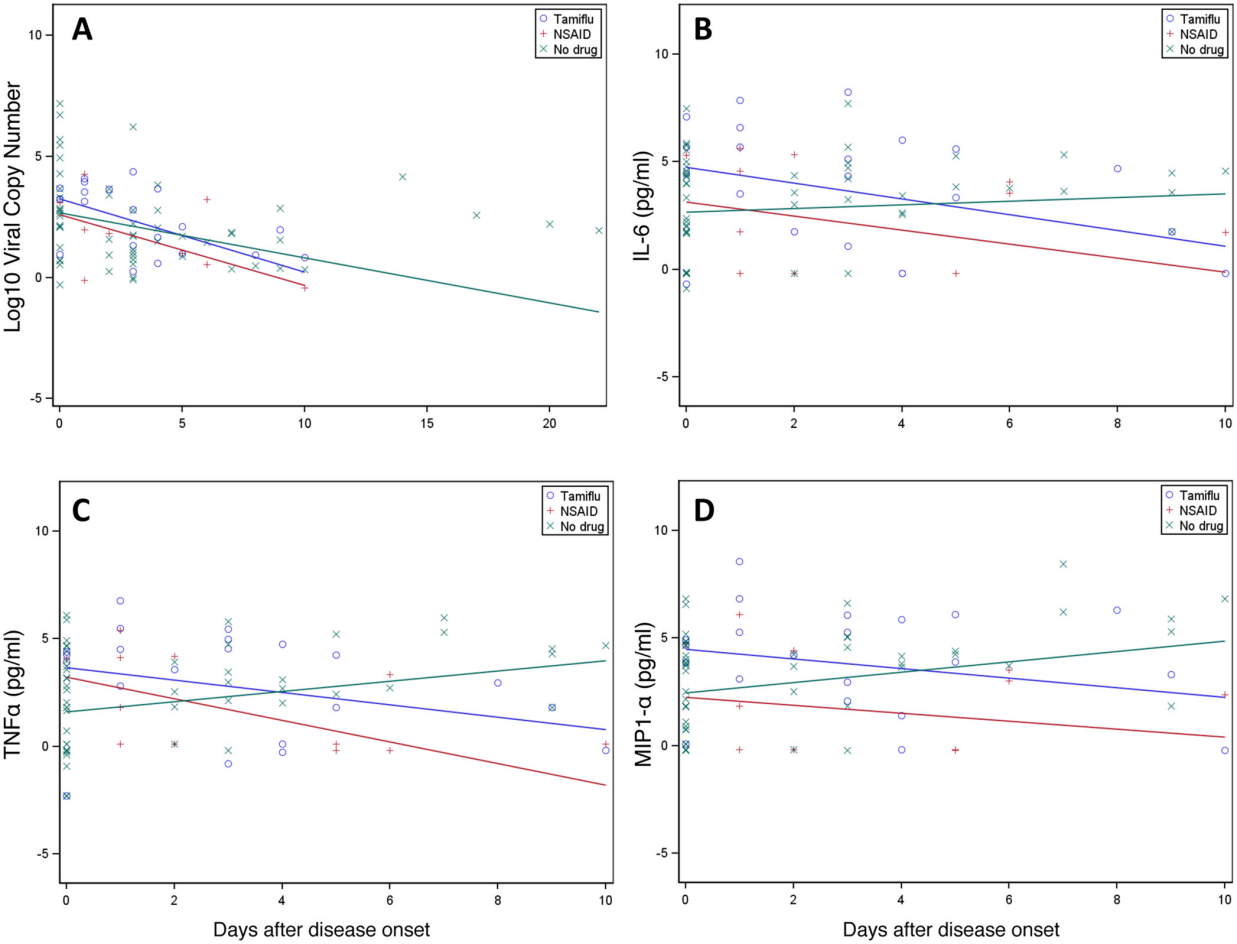
Oseltamivir therapy in longitudinal human samples. (A) Viral loads from nasal swabs and (B-D) natural log-transformed cytokine concentrations from nasal lavages of patients infected with influenza viruses over the course of the 2009–2012 influenza seasons were obtained by (A) PCR or (B-D) Milliplex bead array. Observed measures were presented overlaid with coloured lines that were predicted from LMMs.

We first compared the viral loads of all three groups. Patients on medication were normalized to the start date of the medication. As demonstrated in Fig 3A, and similar to our mouse data (Fig 1C), while there was a trend for an accelerated decline in viral loads in patients on oseltamivir when compared to patients reporting no medications, this difference was not significant and appeared comparable to patients on NSAIDs.

### Oseltamivir treatment is associated with significant reductions in human airway inflammation

Based on our analyses in animal models, we also assessed whether oseltamivir had an effect on cytokine levels in the airways of infected individuals. Adjusting the analysis for viral titre and examining 42 individual cytokines, we found that 24 cytokines were significantly different in oseltamivir-treated individuals. In all cases, oseltamivir treatment resulted in greater reductions in the measured cytokines than in the untreated group (Table 1). Importantly, these cytokines included TNF, MIP1α, and IL-6 (Fig 3B–3D), as had been observed in mice (Fig 3A–3J). Thus, our data from both mice and human samples provide clear evidence that oseltamivir leads to less severe inflammation-associated morbidity, but not a significant reduction in the viral load per se.

**Table 1:** Statistically significant cytokines with oseltamivir-associated reductions in the nasal wash, adjusted by viral loads.

Cytokine	P-value
IL-1β	0.0005
TNF-α	0.0008
IL-1Ra2	0.0012
IL-1a	0.0014
TGF-a	0.0015
EGF	0.0022
IL-8	0.0032
IL-7	0.0038
MIP-1β	0.0052
GRO	0.0058
PDGFAA	0.0066
PDGFABBB	0.0101
Flt3_Ligand	0.0118
IL-12p40	0.0137
GMCSF	0.0147
MIP-1α	0.0172
IL-3	0.0195
IL-12p70	0.0222
sCD40L	0.0258
VEGF	0.0265
IL-6	0.0289
IP10	0.0317
IL-15	0.0370
IL-10	0.0371

### Prophylactic oseltamivir treatment in ferrets reduces influenza-induced morbidity but does not reduce viral load

The effect of oseltamivir prophyalxis was determined in ferrets infected with an H1N1pdm09 virus. At d2 post-infection, we observed that although viral load was moderately higher in oseltamivir-treated ferrets than in untreated ferrets (S1A Fig), ferrets that received oseltamivir displayed reduced morbidity signs. Compared to pre-infection measurements, oseltamivir-treated ferrets had significantly less weight loss than untreated ferrets (S1B Fig). In addition, nasal wash samples from oseltamivir-treated ferrets contained significantly lower total cell numbers and reduced protein concentrations (S1C and S1D Fig).

### Prophylactic oseltamivir treatment reduces the magnitude of virus-specific effector CD8^+^ T cell responses without affecting their functionality

**Fig 4:**
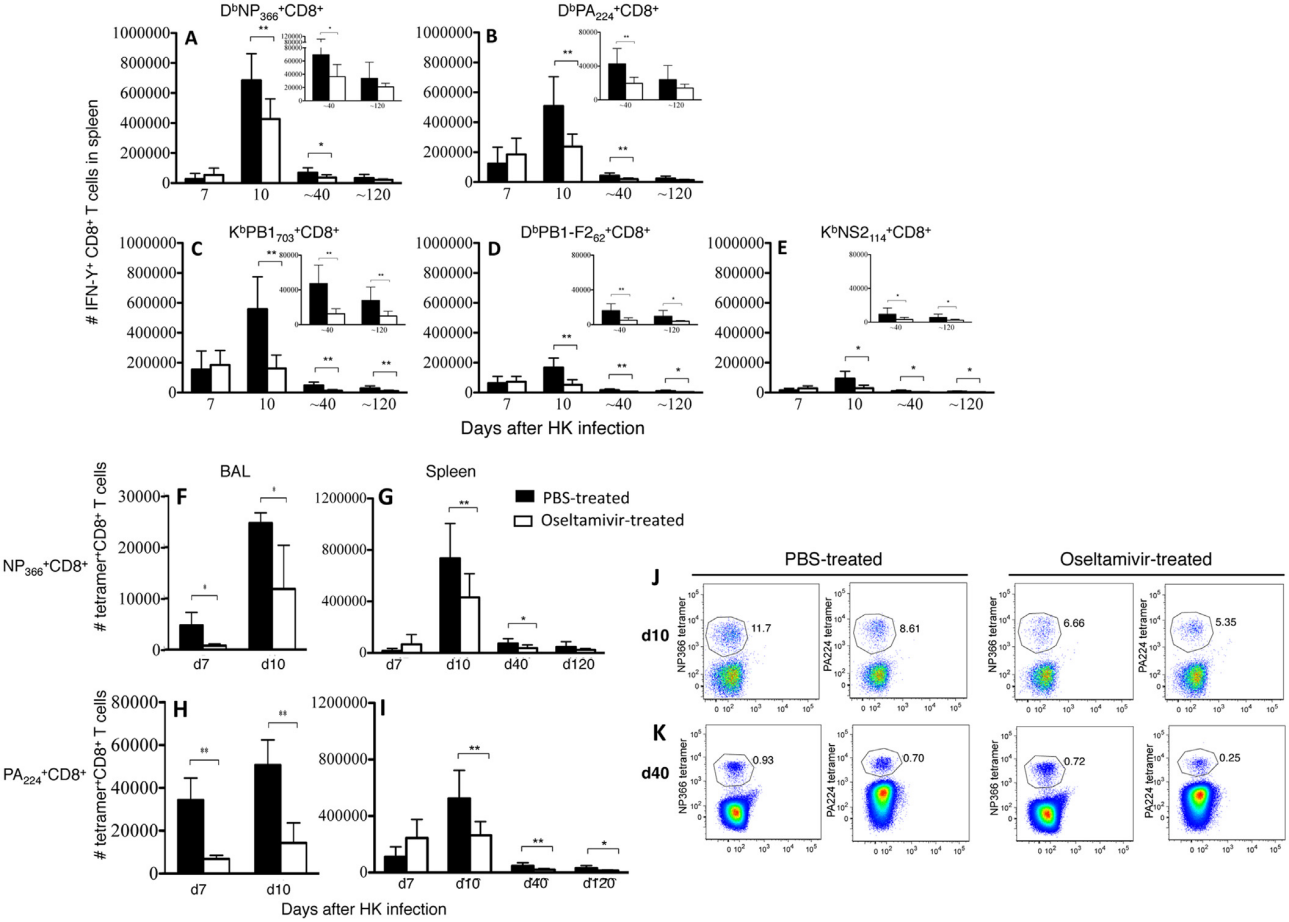
Oseltamivir treatment decreases the magnitude of the effector influenza-specific CD8^+^ T cell responses. BL/6 mice administered with oseltamivir or PBS were assessed for CD8^+^ T cell responses to the influenza A viral epitopes (A) D^b^NP_366_, (B) D^b^PA_224_, (C) K^b^PB1_703-711_, (D) D^b^PB1-F2_62-70_ and (E) K^b^NS2_114-121_ by intracellular IFN-γ staining after a five hour stimulation of splenocytes with cognate peptide. Data represent 9–10 mice per group, analysed over two independent experiments. Epitope-specific CD8^+^ T cells directed at (FG) D^b^NP_366_, (HI) D^b^PA_224_ were enumerated by tetramer staining in both spleen (GI) and at the site of infection (FH). Data represent 9–10 mice per group, analysed over two independent experiments. The mean and standard deviation is shown. (JK) Representative dotplots for the D^b^NP_366_^+^CD8^+^ and D^b^PA_224_^+^CD8^+^ T cell populations on d10 (acute phase) and d40 (memory) are shown; populations are gated on CD8^+^ T cells. The mean and standard deviation is shown. *P<0.05; **P<0.01.

Having established that oseltamivir prophylaxis protects from inflammation-induced morbidity, we asked whether anti-viral treatment affected the generation or function of antigen-specific CD8^+^ T cells. The magnitude of the primary splenic CD8^+^ T cell response against 5 well-characterised H-2^b^-restricted influenza viral epitopes was assessed by ex vivo peptide-pulsed IFN-γ ICS. Enumeration of two immunodominant specificities D^b^NP_366_ and D^b^PA_224_ (Fig 4A and 4B), and three subdominant specificities K^b^PB1_703_, D^b^PB1-F2_62_ and K^b^NS2_114_ (Fig 4C–4E) demonstrated an overall reduction in the magnitude of effector CD8^+^ T cell response in oseltamivir-treated mice. Similar results were obtained by enumeration of influenza-specific CD8^+^ T cells with D^b^NP_366_ and D^b^PA_224_ tetramers at the site of infection (Fig 4F–4H) and spleen (Fig 4G–4I). At d7 and d10 of acute influenza infection, numbers of antigen-specific CD8^+^ T cells in the lungs of oseltamivir-treated mice were significantly reduced compared to PBS-treated controls. These data are in agreement with the diminished recruitment of total CD8^+^ T cells to the site of infection and/or CD8^+^ T cell proliferation (Fig 2K–2N), most likely as a consequence of the observed reduction in cytokine/chemokine concentrations in the lungs of oseltamivir-treated mice (Fig 2).

Given the reduced magnitude of effector cell responses, we asked whether the functionality of the CD8^+^ T cells generated in oseltamivir-treated mice differed inform the PBS-treated controls. One measure of CD8^+^ T cell function is the capacity to produce multiple cytokines [21] following a 5-hour *in vitro* stimulation with cognate peptide. Intracellular cytokine staining of NP_366_- and PA_224_-stimulated splenocytes showed similar frequencies of IFN-γ/TNF-α and IFN-γ/IL-2 (S2 Fig) cytokine-producing CD8^+^ T cells between oseltamivir-treated and PBS-treated mice. Similar results were obtained for less prominent K^b^PB1_703_, D^b^PB1-F2_62_ and K^b^NS2_114_ CD8^+^ T cell responses (data not shown). Overall, oseltamivir treatment of mice reduced the primary influenza-specific CD8^+^ T cell effector response, most likely due to the reduced inflammatory milieu at the site of infection, although, the polyfunctional quality of these cells was unaffected.

### Establishment of functional influenza-specific CD8^+^ T cell memory pools capable of recall responses following the secondary challenge

Despite markedly reduced CD8^+^ T cell responses during the acute phase of influenza infection (d7 and d10), we found that memory CD8^+^ T cell pools were established after anti-viral treatment (d40 and d120). Short term-memory (d40) splenic CD8^+^ T cell responses generated in the oseltamivir-treated group remained significantly reduced compared to PBS-treated mice with an ‘uninterrupted’ influenza infection. Longer-term memory (d120) splenic CD8^+^ T cell memory pools directed against immunodominant D^b^NP_366_ and D^b^PA_224_ epitopes in oseltamivir-treated mice were comparable to those found in PBS-treated group (Fig 4). However, significantly smaller CD8^+^ T cell memory pools were observed for less prominent epitopes K^b^PB1_703_, D^b^PB1-F2_62_ and K^b^NS2_114_ on d120 after primary infection (Fig 4C–4E).

**Fig 5:**
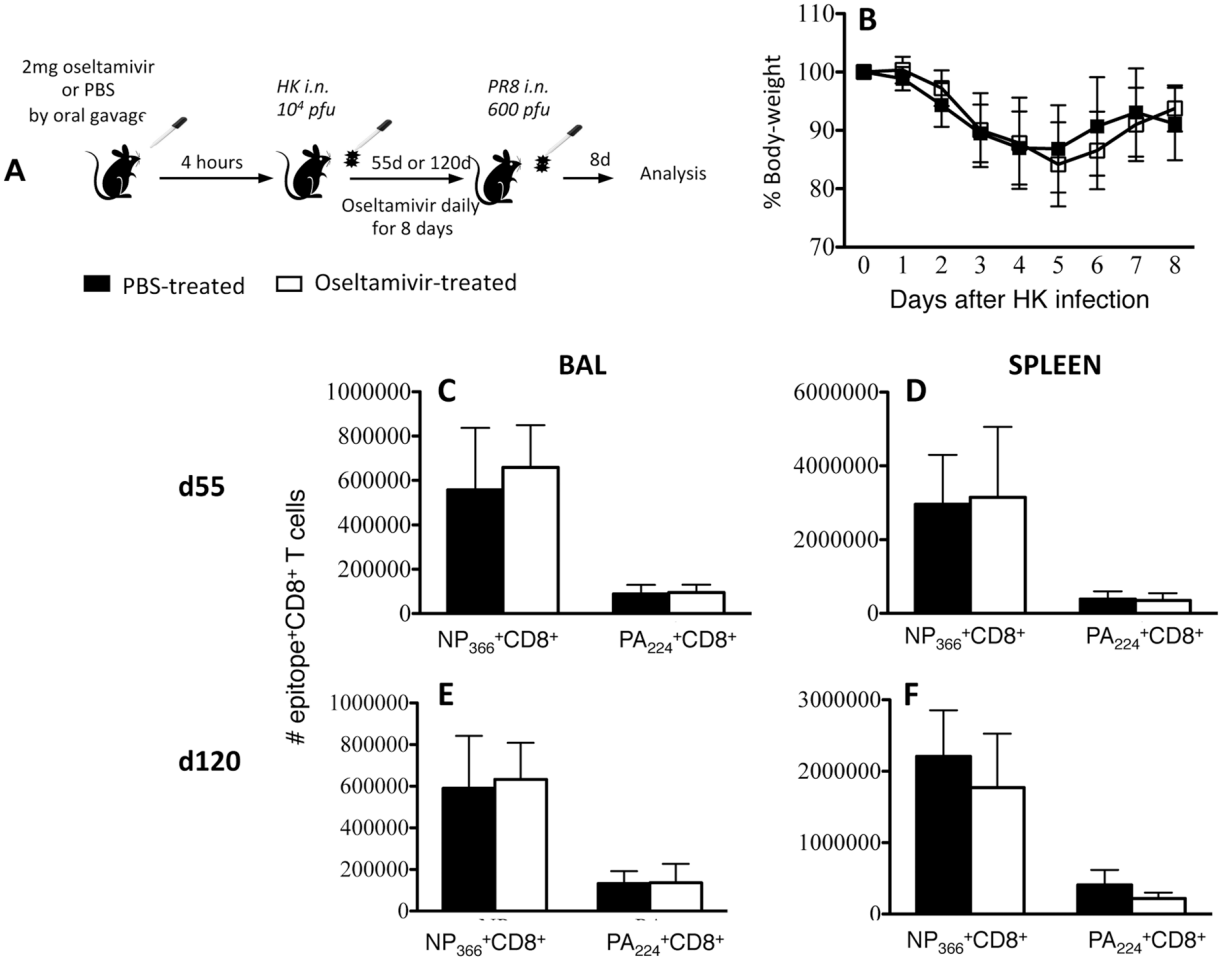
Recall CD8^+^ T cell responses are not compromised in mice with oseltamivir-interrupted primary influenza infection. (A) Naïve female BL/6 mice were administered either oseltamivir or PBS four hours prior to intranasal infection with 10^4^ pfu of HK and then once daily for eight days. Mice were secondarily challenged i.n. with 600 pfu of PR8 either (CD) 55 or (EF) 120 days after primary infection. (B) Mice were monitored daily for body weight loss. D^b^NP_366_ and D^b^PA_224_-specific CD8^+^ T cells in (CE) BAL and (DF) spleen were enumerated by intracellular staining of IFN-γ after five hours of stimulation with NP_366_ or PA_224_ peptide. Data represent the mean and standard deviation of a single experiment with 4–5 mice per group. Similar results were observed from two further recall experiments at day 55 after primary infection.

To understand the recall capacity of memory CD8^+^ T cells established during an influenza infection ‘interrupted’ by oseltamivir-treatment, mice were subsequently secondarily challenged with a distinct influenza strain, PR8 (H1N1). Mice treated with either PBS or oseltamivir during the primary HK (H3N2) influenza infection were challenged after either 55 or 120 days with H1N1-PR8 influenza virus and CD8^+^ T cell responses were analysed 8 days later (Fig 5A).

Recalled D^b^NP_366_, D^b^PA_224_, K^b^PB1_703_, D^b^PB1-F2_62_ and K^b^NS2_114_-specific CD8^+^ T cells from mice treated with either oseltamivir or PBS during the primary infection were enumerated by IFN-γ production. Although oseltamivir-treated mice established reduced influenza-specific memory CD8^+^ T cell pools, especially at the early memory (d40) time-point, these cells produced a recall response of similar magnitude to the mice that had been treated with PBS during the primary infection. This was evident both at the site of infection and in the spleen (Fig 5C–5F, S4 Fig). Furthermore, secondary D^b^NP_366_- and D^b^PA_224_-specific CD8^+^ T cells from oseltamivir-treated mice showed a similar polyfunctionality profile to CD8^+^ T cells recovered from mice treated with PBS during primary influenza infection (S3 Fig). Weight-loss was also comparable between oseltamivir- and PBS-treated mice, demonstrating a similar disease severity between the groups after the secondary infection (Fig 5B). Additionally, both animal groups cleared the virus by d8 of secondary infection (data not shown).

Thus, our data show that CD8^+^ T cell memory pools established in mice treated with anti-viral prophylaxis during the primary influenza infection were fully functional and capable of normal recall capacity after secondary challenge.

### Generation of influenza-specific CD4^+^ T cell responses and antibodies in presence of oseltamivir prophylaxis

**Fig 6:**
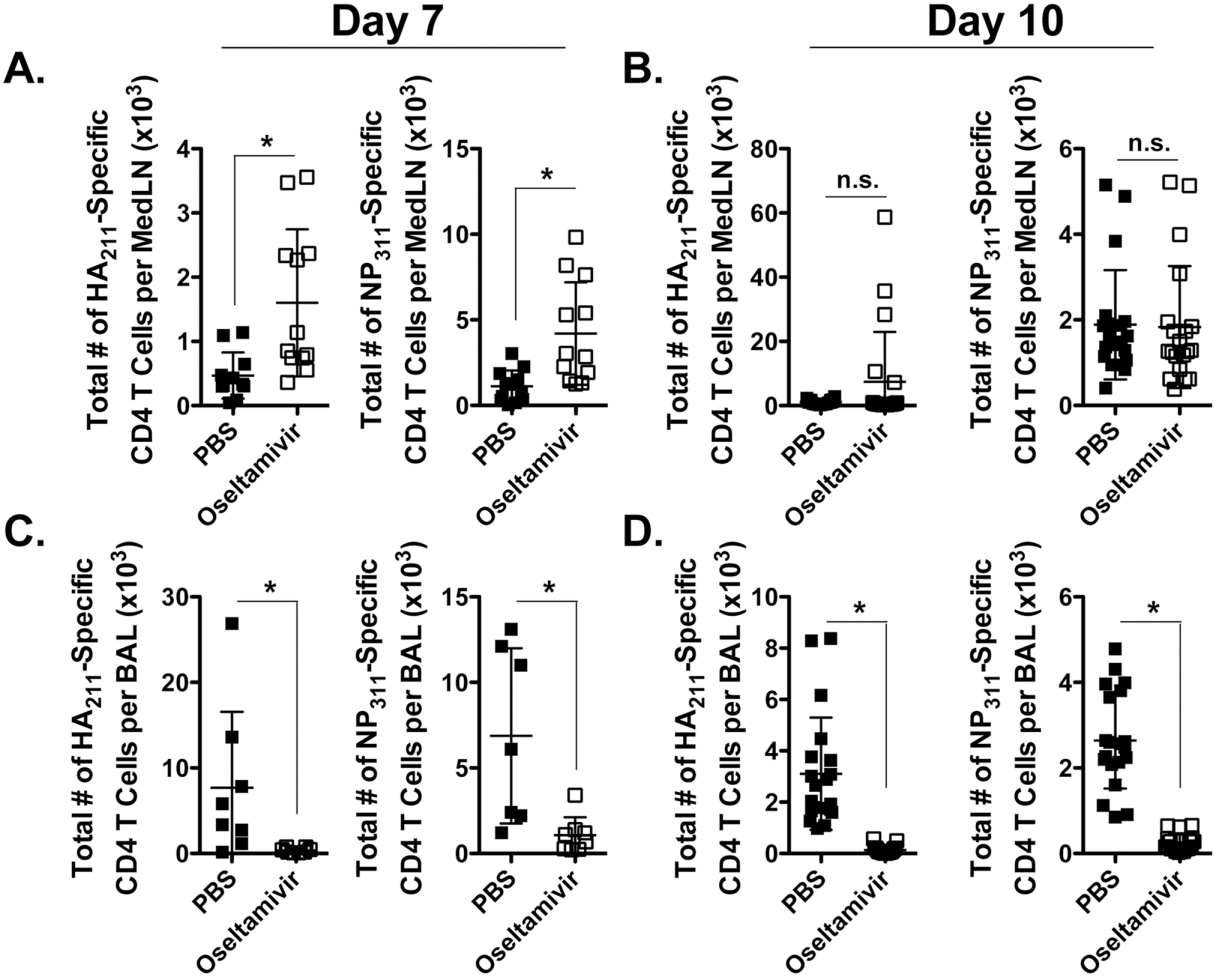
Influenza-specific CD4^+^ T cell responses in mice prophylactically treated with oseltamivir. Mice were treated with PBS or oseltamivir and infected with influenza virus X31 as in Fig 1A. At (AC) 7 and (BD) 10 days post-infection, the mediastinal LNs (MedLN) and BAL fluid were harvested and assessed for the total number of influenza IA^b^-HA_211_ and NP_311_-specific CD4^+^ T cells by intracellular cytokine staining after *ex vivo* stimulation. These data are from 8–13 mice (2 individual experiments) at day 7 p.i and from 20 mice (4 individual experiments) at day 10 p.i. Error bars represent standard error of the mean. * *p*<0.05. n.s., not significantly different.

To extend the findings beyond anti-viral CD8^+^ T cell responses, we assessed CD4^+^ T cell responses and antibody levels at different stages of influenza virus infection in mice treated with PBS or oseltamivir. CD4^+^ T cell responses to two epitopes (HA_211_ and NP_311_) were analysed. Firstly, we examined whether virus-specific CD4^+^ T cell responses were also diminished in the lung draining LNs (mediastinal LNS, MedLN) and at the site of infection in mice prophylactically-treated with oseltamivir as compared to controls. Interestingly, early after infection, at d7, there was an increase (*p*<0.05) in the total number of I-A^b^HA_211_ and I-A^b^NP_311_-specific CD4^+^ T cells in the MedLN of oseltamivir-treated mice as compared to controls, where at d10, virus-specific CD4^+^ T cell numbers were equivalent in the MedLN (Fig 6A and 6B). However, similar to the virus-specific CD8^+^ T cell response, virus-specific CD4^+^ T cell numbers were also reduced (*p*<0.05) in the BAL at both 7 and 10 days in oseltamivir-treated mice as compared to PBS controls (Fig 6C and 6D). Together, these data suggest that recruitment/proliferation of the virus-specific T cell response to the site of infection is impaired by oseltamivir treatment.

**Fig 7:**
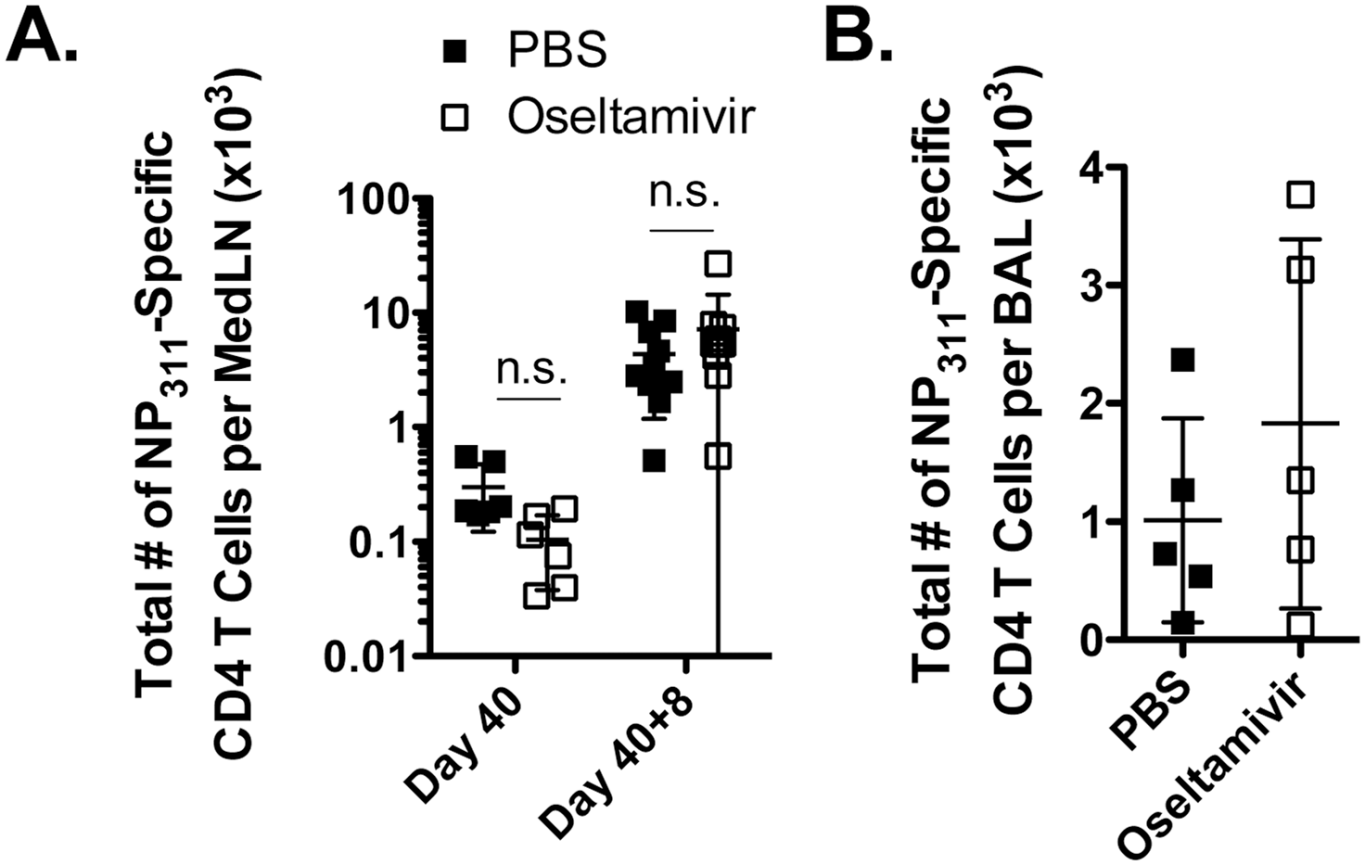
Normal influenza-specific CD4^+^ T cell responses in oseltamivir-treated mice after secondary infection. Mice were treated with PBS or oseltamivir and infected with influenza virus X31, then rested for 40 days. At this time point, mice were infected i.n. with 600 pfu of influenza PR8. At 8 days post-secondary infection, (A) MedLN and (B) BAL fluid were harvested and assessed for the total number of influenza IA^b^- NP_311_-specific CD4^+^ T cells by intracellular cytokine staining after *ex vivo* stimulation. These data represent 2 individual experiments with 4–5 mice per group. Error bars represent standard error of the mean. n.s., not significantly different.

We then examined the maintenance of influenza-specific CD4^+^ T cell memory in oseltamivir and PBS-treated mice after infection and found similar total numbers (*p*>0.05) of I-A^b^HA_211_ and I-A^b^NP_311_-specific CD4^+^ T cells in the MedLN at d40 (Fig 7A). After secondary infection, d8, we also observed similar numbers (*p*>0.05) of both I-A^b^HA_211_ and I-A^b^NP_311_-specific CD4^+^ T cells in the MedLN (Fig 7A) and in the BAL (Fig 7B). The polyfunctionality of the recall CD4^+^ T cell responses were also similar in oseltamivir-treated and untreated mice (data not shown).

**Fig 8:**
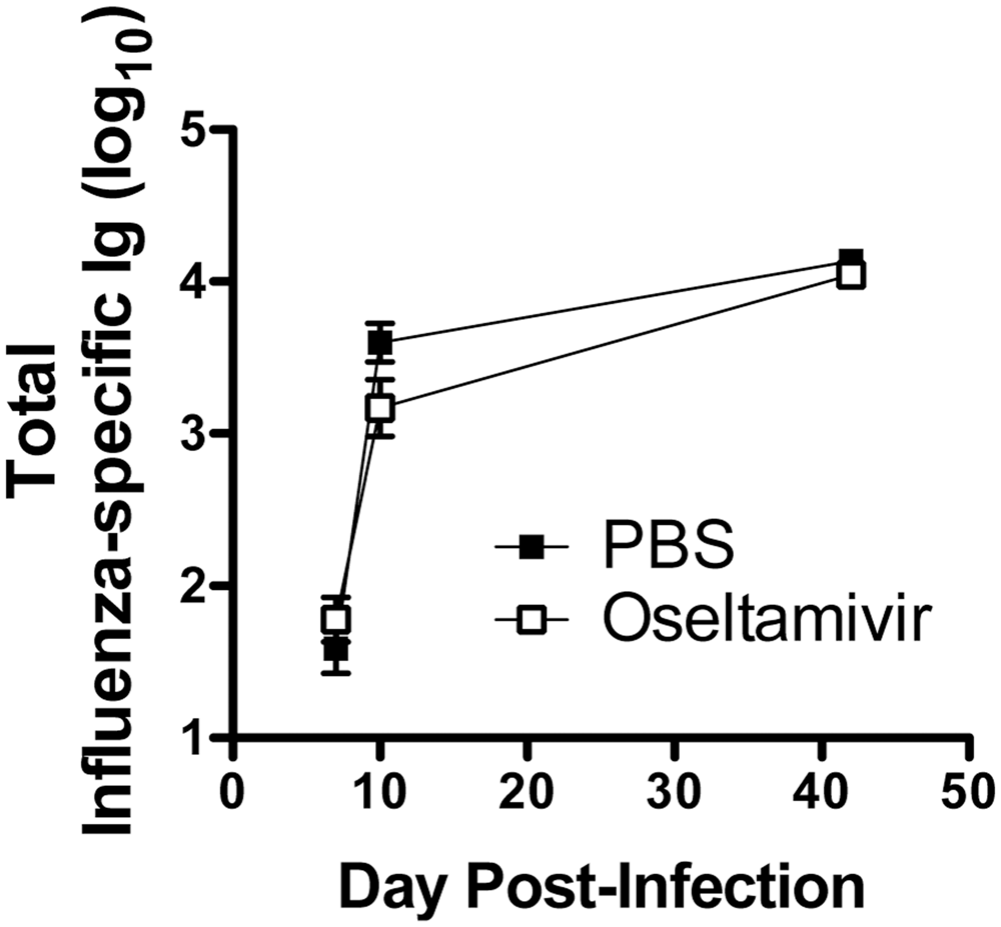
Oseltamivir-treated mice develop normal antibody responses after influenza infection. Mice were treated with PBS or oseltamivir, then infected with X31. At 7, 10 and 42 days post-infection, serum was collected and assessed for total influenza X31-specific antibody by ELISA. Error bars represent standard error of the mean of 5–10 mice for each time point.

Additionally, the total influenza virus-specific Ig response in sera was also analysed and the results showed comparable Ig levels in oseltamivir-treated and-untreated mice at days 7, 10 and 40 (Fig 8). These findings suggest that both CD8^+^ and CD4^+^ T cell memory and antibodies can be established while taking oseltamivir prophylaxis.

## Discussion

Memory CD8^+^ T cells directed toward more conserved internal proteins of influenza virus can provide cross-strain immunity [8,22–24]. Whilst the presence of such memory CD8^+^ T cells cannot prevent influenza re-infection, pre-existing CD8^+^ T cells diminish disease severity and promote more rapid recovery, manifested by milder symptoms, decreased virus shedding and less transmission [25,26]. Furthermore, pre-existing CD8^+^ T cell memory pools provide critical protection in the face of a pandemic viral strain, to which pre-existing antibody responses are minimal [11,12,14–16]. As such, the generation and maintenance of influenza-specific CD8^+^ T cell memory is of great importance, the lack of which is a major limitation of the current influenza vaccine.

**Fig 9:**
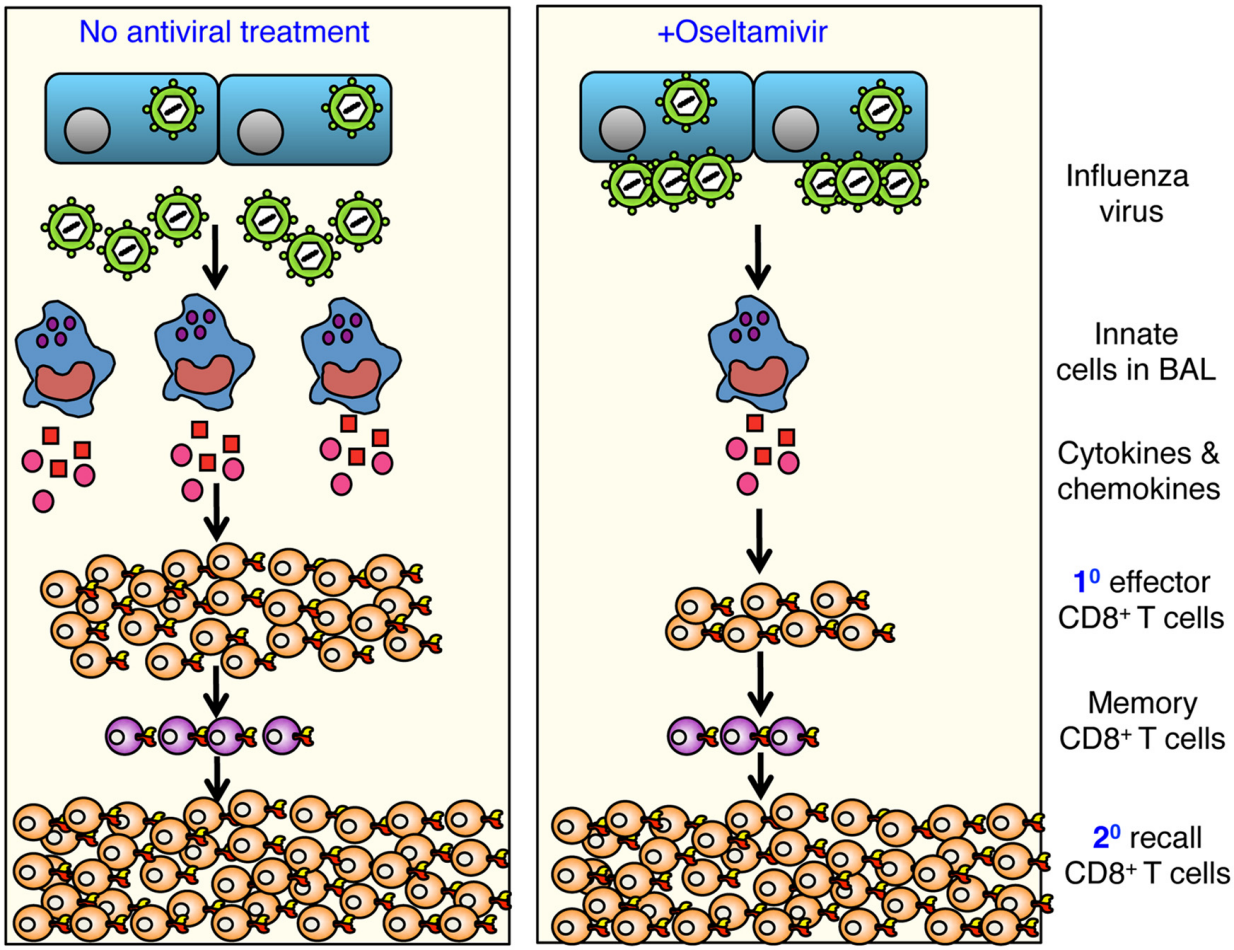
Oseltamivir prophylaxis reduces inflammation-induced morbidity while maintaining establishment of functional cross-strain protective CD8^+^ T cell memory pools. Oseltamivir prophylaxis of influenza virus-infected mice results in reduced recruitment of innate cells to the airways and this correlates with a reduced cytokine/chemokine inflammatory milieu. In addition, oseltamivir treatment reduced, the magnitude of influenza-specific effector CD8^+^ T cell responses. However, functional memory CD8^+^ T cell pools were established effectively in oseltamivir-treated mice, and these cells could elicit recall responses of similar magnitude and quality to those generated in PBS-treated control animals. This provides evidence that long-term memory T cells can be generated during an oseltamivir-interrupted infection.

Given the dependence on influenza antivirals in the midst of a pandemic, an important question is how the administration of such antivirals affects the establishment of immunological CD8^+^ T cell memory. Our study dissected establishment and function of influenza-specific CD8^+^ T cell memory generated during an influenza infection ‘interrupted’ by treatment of mice with the viral neuraminidase inhibitor, oseltamivir. Our data in mice, ferrets and humans demonstrate that oseltamivir prophylaxis greatly reduces morbidity, which correlates with diminished recruitment of cells to the site of infection, and reduced production of inflammatory cytokines and chemokines. Furthermore, the effector CD8^+^ T cell response to five well-characterised influenza epitopes was reduced in both lungs and spleen during the acute phase of the infection. Despite this, fully functional memory CD8^+^ T cell pools capable of cross-strain reactivation following infection with a distinct influenza subtype were established (Fig 9). Our study is the first to show that in the case of an influenza pandemic, while prophylactic oseltamivir treatment may limit virus growth and reduce disease severity, the capacity to generate memory CD8^+^ T cells specific for the newly emerged virus is not compromised.

Similar to previous studies [27], the administration of oseltamivir during influenza A virus infection ameliorated the disease in mice. As weight-loss is indicative of morbidity, mice administered oseltamivir during influenza virus infection experienced a less severe disease course. The reduction in total cytokines and chemokines assayed at the site of infection in mice administered oseltamivir is in agreement with these observations, as influenza virus-associated morbidity has been related to innate immune-induced pneumonia and the cytokine storm [28].

While reduced morbidity was observed earlier after infection in oseltamivir-treated mice, surprisingly, only modest differences of 0.5 log were detected in lung virus titres between treated and mock-treated mice at day 3 post-infection. Similarly, ferrets treated with oseltamivir prior to influenza infection did not demonstrate a significant reduction in viral load, the same finding as reported in our previous study [29]. Previously published data both *in vivo* [30] and *in vitro* [31] demonstrate the ‘sequestration’ of virus as a mechanism of action of oseltamivir. It may be that the formation of viral foci in the presence of oseltamivir treatment within the lung leads to a difference in the way the innate immune system ‘sees’ and responds to the virus.

However, despite high viral load early in the influenza infection in oseltamivir-treated mice, there was a significant reduction in the cellular infiltrate to the site of infection, reflecting the diminished global cytokine and chemokine millieu at the site of infection. Such reduced innate responses reflect a difference in the way the host ‘sees’ viral antigen in the presence of oseltamivir. Together, our findings show diminished innate response to influenza infection due to the viral load being ‘sequestered’ in the presence of oseltamivir and therefore not effectively activating the immune response.

Mice treated with oseltamivir prior to and during influenza infection had a reduced primary CD8^+^ T cell response to the epitopes D^b^NP_366-374_ and D^b^PA_224-233_ as well as the less prominent K^b^PB1_703-711_, D^b^PB1-F2_62-70_ and K^b^NS2_114-121_, both at the site of infection and in the spleen. These results may reflect the reduced number of APCs at the site of infection and hence diminished antigen presentation at both d3 and d7 post-influenza virus infection. Alternatively, the diminished CD8^+^ T cell response to virus may also be due to reduced recruitment of naïve CD8^+^ T cells into the adaptive response, recruitment and/or proliferation of CD8^+^ T cells to the site of infection, or activation status of CD8^+^ T cells. Additional experiments would be necessary to distinguish between these possibilities.

Importantly, a functional memory CD8^+^ and CD4^+^ T cell pools capable of eliciting recall responses to the same extent as following the normal, ‘uninterrupted’, influenza infection, was established during this drug-reduced effector phase. These results provide important insights into the development of memory T cells in response to influenza virus infection. Our data are in agreement with previous elegant experiments, which used antibiotic treatment prior to *Listeria monocytogenes* [32] to show that the full expansion to effector status is not a pre-requisite for the generation of long-term memory CD8^+^ T cells. Similarly, our previous experiments utilizing clonal dissection of influenza-specific CD8^+^ T cells at different stages of infection [17,33] or transfer of influenza-specific CD8^+^ T cells recovered at different days after influenza virus infection [18] suggested the early establishment of influenza-specific CD8^+^ T cell memory pools. The present study, however, provides direct evidence that influenza-specific CD8^+^ T cell memory can be established during anti-viral prophylaxis. Furthermore, as shown by the published reports, the cytokines important for T cell memory generation include IL-2 and IL-12 [34,35]. Interestingly, both IL-2 and IL-12 were unaffected by the oseltamivir prophylaxis in our study, suggesting that the necessary signals for CD8^+^ T cell memory generation are available.

Influenza severity can be controlled by antivirals such as oseltamivir, if administered early in the infection. In case of an influenza pandemic, such as the 2009-H1N1 pandemic, or emergence of a new influenza strain like the recent H7N9, oseltamivir is also used as a prophylaxis for those in contact with influenza-infected patients. Oseltamivir treatment represents an ‘interrupted’ course of influenza virus infection with ameliorated disease that still allows for functional memory CD8^+^ T cell establishment. Thus, our study contributes to the understanding on how oseltamivir prophylaxis affects influenza-induced morbidity versus immune responses, and provides novel insights into establishment of protective immune responses, important for the new vaccine and drug therapies.

## Materials and Methods

### Ethics statement

All animal experimentation was conducted following the Australian National Health and Medical Research Council Code of Practice for the Care and Use of Animals for Scientific Purposes guidelines for housing and care of laboratory animals and performed in accordance with Institutional regulations after pertinent review. Mice experiments were approved by the University of Melbourne Animal Ethics Experimentation Committee in Melbourne under the project 0810974.2.

Experiments using ferrets was conducted with approval from the Commonwealth Serum Laboratories Limited / Pfizer Animal Ethics Committee (project license number 868) in strict accordance with the Australian Government, National Health and Medical Research Council Australian code of practice for the care and use of animals for scientific purposes (8^th^ edition). Animal studies were conducted at CSL Limited using services provided under a Support Service Agreement between CSL Limited and WHO Collaborating Centre for Reference and Research on Influenza. Experiments were not conducted at Pfizer or supported by Pfizer.

H1N1 cohort study was conducted in compliance with 45 CFR 46 and the Declaration of Helsinki. Institutional Review Boards of St. Jude Children's Research Hospital and the University of Tennessee Health Science Center / Le Bonheur Children's Hospital approved the study. Written, informed consent was obtained from participants' parents/guardians as well as written assent from age-appropriate subjects at the time of enrollment.

### Oseltamivir treatment and viral infection of mice

Female C57BL/6 (H-2^b^) mice were bred and housed in the Animal Facility, Department of Microbiology and Immunology at University of Melbourne. Isofluorane-anaethetised (Veterinary Companies of Australia Pty Ltd, NSW, Australia) mice were administered 100μl of PBS either alone or supplemented with 20 mg/ml oseltamivir phosphate (kindly provided by Hoffmann-La Roche Ltd., Basel, Switzerland) by oral gavage using a 25.4mm animal feeding needle (Popper and Sons, New Hyde Park, NY, USA) four hours prior to primary infection. This process was repeated once daily for eight days after infection, or until the experimental endpoint. Oseltamivir dose of 100 mg/kg/day was based on previous studies showing that a dose of 100 mg/kg/day resulted in the greatest reduction of morbidity after treatment with oseltamivir [36].

B6 mice were intranasally (i.n.) infected with 1x10^4^ plaque forming units (pfu) of HK (H3N2; HK-X31) in 30μl of PBS. For recall, mice primed with HK were challenged i.n. with 600 pfu of PR8 at least 4 weeks later. The PR8 and HK viruses differ in their surface hemagglutinin and neuraminidase glycoproteins (H1N1 and H3N2, respectively), but share the PR8 internal components (NP, PA, M1, PB1, PB1-F2 and NS2). Mice were lightly anaethetised prior to viral infection and body-weight of mice was monitored daily. Mice were culled by CO_2_ asphyxiation at the experimental endpoint. Virus stocks were grown in the allantoic cavity of 10 day-old embryonated chicken eggs, and the viral titre was determined by a plaque assay on MDCK monolayers.

#### Tissue sampling of mice

Spleens were taken and pressed through 70μm nylon sieves (BD Falcon, NJ, USA). B cell depletion was performed by incubation of splenocytes for 45 mins at 37°C, 5% CO_2_ on plates coated with 200μg/ml goat anti-mouse anti-IgM/IgG (Jackson ImmunoResearch Labs, USA). Bronchoalveolar lavage (BAL) was performed to sample cells from the respiratory tract and lungs. Lungs were homogenised by Polytron System PT 1200 CL 230V (Kinematica, Lucerne, Switzerland).

#### Determination of viral titres by plaque assay

MDCK monolayers were infected with lung homogenates, or the virus, at varying concentrations for 45 mins at 37°C, 5% CO_2_ before the addition of an Agarose/L15 or MEM overlay containing Trypsin Worthington (Worthington Biochemical, NJ, USA). Plates were incubated at 37°C, 5% CO_2_ for 3 days before plaques were counted.

#### Cytometric Bead Analysis

The CBA flex set (BD Bioscience) was used as per manufacturer’s instructions to determine the cytokine concentrations of BAL supernatant. Data were acquired using FACSCantoII and analysis was by FCAP Array software (Soft Flow Inc., Pecs, Hungary).

#### Cellular staining of BAL

BAL cells were stained separately with two antibody cocktails. The first was for NK cells with NK1.1-PE (BD PharMingen), T cells with TCRβ-APC (BD PharMingen) and NKT cells NK1.1-PE and TCRβ-APC. The second stain was for neutrophils with Ly6G-FITC (BD PharMingen) and B cells with B220-PE (Biolegend). NKT cells were identified using CD1dα-GALCER tetramer conjugated to APC. Propidium Iodide was used to exclude dead cells before data were acquired using either FACSCalibur or FACSCantoII.

#### Analysis of the influenza-specific T cell response

Splenocytes and BAL cells were stained with PE- or APC-conjugated tetrameric complexes of the H-2D^b^ or H-2K^b^ MHC class I glycoprotein and the influenza viral peptides NP_366-374_ (ASNENMETM), PA_224-233_ (SSLENFRAYV), PB1_703-711_ (SSYRRVPGI), PB1-F2_62-70_ (LSLRNPILV) or NS2_114-121_ (RTFSFQLI) for 1hr in the dark at room temperature. Cells were then stained with anti-CD8-FITC or–Pacific Blue at 4°C for 30 minutes and data acquired by FACSCalibur or FACSCantoII. Intracellular cytokine staining was performed by incubation of cells with viral peptide for 5hrs in the presence of IL-2 at 37°C, 5% CO_2_ before surface staining with either anti-CD8-PerCPCy5.5 or–Pacific Blue. Cells were then fixed, permeabilised and intracellularly stained with IFN-γ-FITC, TNFα-APC and IL-2-PE. Data were acquired on FACSCalibur or FACSCantoII. Influenza-specific CD4^+^ T cell responses were analysed as previously described [37].

#### Analysis of the influenza-specific levels

Flat bottom well polyvinyl plates were coated with recombinant HA protein derived from X31 (5μg/ml) in PBS for 18–20 hours at room temperature in a humidified atmosphere. The antigen was removed and BSA (10mg/ml) in PBS added for 1 hr before washing with PBS containing v/v 0.05% Tween-20 (Aldrich, Milwaukee, USA). Serial dilutions of sera obtained from infected mice were added to wells and held overnight at room temperature. After washing, bound antibody was detected using horseradish peroxidase-conjugated rabbit anti-mouse IgG antibodies (Dako, Glostrup, Denmark) in conjunction with enzyme substrate (0.2 mM 2,2’-azino-bis 3-ethylbenzthiazoline-sulfonic acid in 50 mM citric acid containing 0.004% hydrogen peroxide). The reaction was stopped by addition of 50 ml of 0.05M NaF. The titers of antibody are expressed as the reciprocal of the highest dilution of serum required to achieve an optical density (at a dual wavelength of 405nm and 450nm) of 0.2.

### Oseltamivir treatment and infection of ferrets

Ferrets (n = 4 in each group; source: outbred from multiple breeders) were anaesthetized [50:50 mix of Ketamine (100 mg/mL): Ilium Xylazil (Xyalazine; 20 mg/mL)] and infected by intranasal inoculation with 10^5^ TCID_50_ (median tissue culture infectious dose) of MDCK (Madin-Darby canine kidney (MDCK; ATCC CCL-34)-propagated A/Perth/265/2009 A(H1N1)pdm09 influenza virus (2.0 x 10^6^ TCID_50_/mL). For oseltamivir treatment, ferrets were orally given 5 mg/kg oseltamivir phosphate (kindly provided by Hoffmann-La Roche Ltd., Basel, Switzerland) two hours prior to infection and then twice daily post-infection. Body temperature, weight and nasal washes of all ferrets were collected at day 2 post-infection. Virus titer, cell concentration and protein concentration of nasal washes were measured as described [38]. Oseltamivir treated and untreated ferrets were nasal washed on day 2 post-infection to determine viral load, cell count and nasal wash protein concentration. Ferrets were housed individually in high efficiency particulate air filtered cages with ad libtum to food, water and enrichment toys. Ferrets were sacrificed by intramuscular injection of anaesthesia [50:50 mix of Ketamine (100 mg/mL): Ilium Xylazil (Xyalazine; 20 mg/mL)] followed by an overdose of pentobarbitone sodium (Lethabarb; 0.5 mL/kg).

### H1N1 patient cohort

Written, informed consent was obtained from participants' parents/guardians as well as written assent from age-appropriate subjects at the time of enrollment. Eligible individuals were those with acute respiratory illness meeting the case definition for influenza (fever or feverishness accompanied by cough or sore throat) who were symptomatic for 96 hours or less, and their household contacts. Influenza virus A or B diagnosis was confirmed by detection of matrix gene (influenza A) or nonstructural NS1 gene (influenza B) by quantitative real time reverse transcription (RT)-PCR. The majority of donors belong to the household cohort described in our earlier study [20]. The numbers of participants and their ages were: (1) ‘no drug’ group with 52 participants, age 0.24–50.8 yrs, average age 14.6, 44.2% males; (2) ‘oseltamivir’ group with 14 participants, age 0.14–40.6 yrs, average age 9.09, 42.6% males; (3) ‘NSAID’ group with 9 participants, age 0.33–29.9 yrs, average age 9.91, 44.4% males.

The study was purely observational. The patients were given oseltamivir by their physicians as part of standard-of-care treatment. The non-drug treated cases were the members of the cohort that reported no use of NSAIDs or antivirals. Index cases were asked to provide nasal swabs, nasal lavages, and blood on the day of enrollment (Day 0) and Days 3, 7, 10, and 28, whereas household contacts were asked to provide nasal swabs on Day 0, 3, 7, and 14 and blood and nasal lavages on Days 0 and 28. Participants were asked to rank their symptom severity daily according to a visual analog scale. Household contacts that tested positive for influenza were enrolled as cases and followed the case-sampling protocol. The Luminex MAP system was used with a MILLIPLEX MAP human cytokine immunoassay (Millipore, St. Charles, MO) for detection of cytokines from samples according to the manufacturer's protocol from the nasal wash and serum samples.

#### Statistics

Statistical analysis was by a two-tail, type two Student’s t-test. Linear mixed effects models (LMMs) were applied in SAS 9.3 [39] to investigate the rate of changes of cytokines across concomitant drug groups, adjusted by viral loads. Measured cytokines were natural log-transformed and viral loads were log_10_ transformed. An LMM was also used to explore the association of concomitant drugs with viral loads. A p-value of 0.05 or less was considered significant, unless otherwise specified.

## Supporting Information

S1 Fig Oseltamivir prophylaxis reduces morbidity, inflammation but not viral titer in influenza A infected ferrets. Ferrets (n = 4 in each group) were infected with 10^5^ Log_10_TCID_50_/mL of influenza A/Perth/265/2009 virus. For treatment group, ferrets were orally given 5mg/kg oseltamivir phosphate two hours prior to infection and twice daily for five days. Nasal wash was collected at day 2 post-infection from all ferrets. (A) Viral titer in nasal wash, (B) body weight (C) cell counts recovered from 1mL of nasal wash and (D) total protein concentration in nasal wash. Data are mean±SEM.
(TIF)

S2 Fig CD8^+^ T cell polyfunctionality is maintained in mice treated with oseltamivir prior to influenza A virus infection. Mice were administered oseltamivir or PBS four hours prior to infection with 10^4^ pfu of HK and then once daily for eight days. Splenocytes were stimulated by NP_366_ or PA_224_ peptide for five hours prior to intracellular staining for IFN-γ, TNFα and IL-2. The proportion of CD8^+^ T cells coproducing IFN-γ and TNFα (AC), or IFN-γ and IL-2 (BD) are shown for the influenza A viral epitopes D^b^NP_366_ (AB), and D^b^PA_224_ (CD). Data represent independent experiments of 4–5 mice at each time-point. Each time-point was repeated and similar results were observed.
(TIF)

S3 Fig Establishment of polyfunctional recall CD8^+^ T cell responses after oseltamivir prophylaxis. Naïve female BL/6 mice were administered either oseltamivir or PBS four hours prior to i.n. infection with 10^4^ pfu of HK and then once daily for eight days. Mice were secondarily challenged i.n. with 600 pfu of PR8 120 days after primary infection. Splenocytes or BAL cells were stimulated with NP_366_ or PA_224_ peptide for five hours prior to intracellular staining for IFN-γ, TNFα and IL-2. The proportion of CD8^+^ T cells coproducing IFN-γ and TNFα (AC), or IFN-γ and IL-2 (BD) are shown for the influenza A viral epitopes D^b^NP_366_ (AB), and D^b^PA_224_ (CD). Data are representative of one experiment of 5 mice per group. Similar results were observed from two further recall experiments at day 55 after primary infection.
(TIF)

S4 Fig Oseltamivir treatment of mice infected with influenza A virus reduces the primary CD8^+^ T cell response, but does not affect recall upon secondary challenge. Naïve female BL/6 mice were administered either oseltamivir or PBS four hours prior to intranasal infection with 10^4^ pfu of HK and then once daily for eight days. Mice were secondarily-challenged intranasally with 600 pfu of PR8 either (AB) 55, or (CD) 120 days after primary infection The less dominant K^b^PB1_703_, D^b^PB1-F2_62_ and K^b^NS2_114_-specific CD8^+^ T cells in both BAL (AC) and spleen (BD) were enumerated by intracellular staining of IFN-γ after five hours of stimulation with cognate peptide. Data represent the mean and standard deviation of a single experiment with 4–5 mice per group and are plotted on the same scale as that of the immunodominant D^b^NP_366_ and D^b^PA_224_ (Fig 6) to demonstrate relative contributions to the secondary response. Similar results were observed from two additional recall experiments at day 55 after primary infection.
(TIF)
