# Supplementary figures and images for: Oseltamivir Prophylaxis Reduces Inflammation and Facilitates Establishment of Cross-Strain Protective T Cell Memory to Influenza Viruses
Source: PLoS One. 2015 Jun 18;10(6):e0129768. doi: 10.1371/journal.pone.0129768 (PMC4473273; doi:10.1371/journal.pone.0129768)

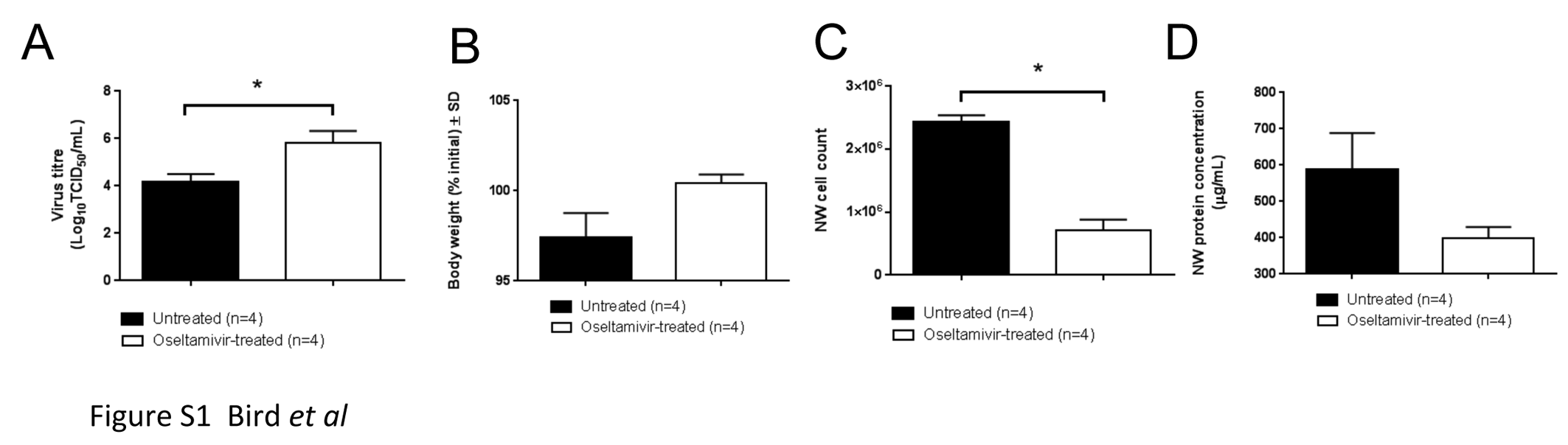

Supplement: S1 Fig — Ferrets (n = 4 in each group) were infected with 105 Log10TCID50/mL of influenza A/Perth/265/2009 virus. For treatment group, ferrets were orally given 5mg/kg oseltamivir phosphate two hours prior to infection and twice daily for five days. Nasal wash was collected at day 2 post-infection from all ferrets. (A) Viral titer in nasal wash, (B) body weight (C) cell counts recovered from 1mL of nasal wash and (D) total protein concentration in nasal wash. Data are mean±SEM. (TIF) [file pone.0129768.s001.tif]

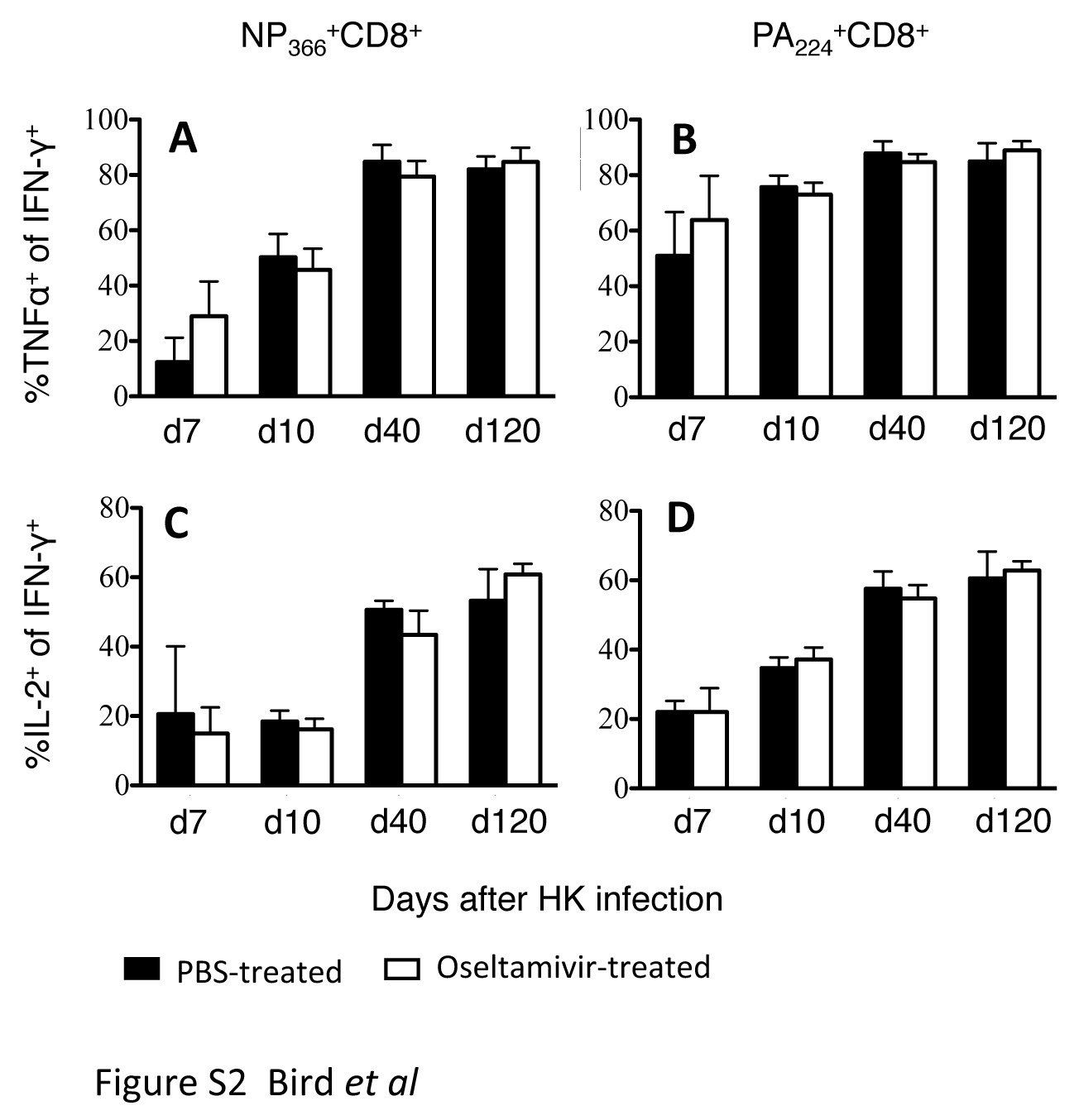

Supplement: S2 Fig — Mice were administered oseltamivir or PBS four hours prior to infection with 104 pfu of HK and then once daily for eight days. Splenocytes were stimulated by NP366 or PA224 peptide for five hours prior to intracellular staining for IFN-γ, TNFα and IL-2. The proportion of CD8+ T cells coproducing IFN-γ and TNFα (AC), or IFN-γ and IL-2 (BD) are shown for the influenza A viral epitopes DbNP366 (AB), and DbPA224 (CD). Data represent independent experiments of 4–5 mice at each time-point. Each time-point was repeated and similar results were observed. (TIF) [file pone.0129768.s002.tif]

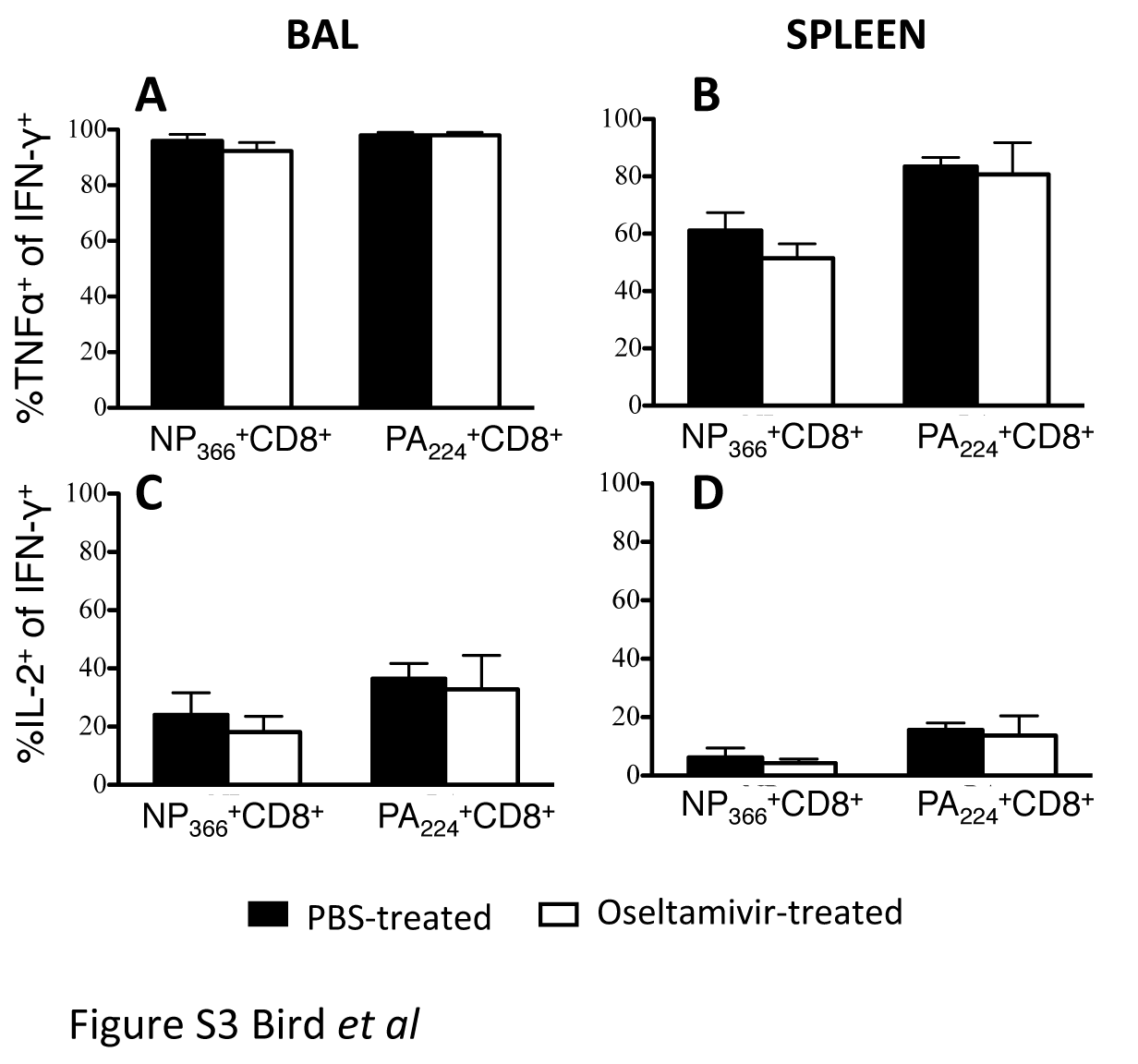

Supplement: S3 Fig — Naïve female BL/6 mice were administered either oseltamivir or PBS four hours prior to i.n. infection with 104 pfu of HK and then once daily for eight days. Mice were secondarily challenged i.n. with 600 pfu of PR8 120 days after primary infection. Splenocytes or BAL cells were stimulated with NP366 or PA224 peptide for five hours prior to intracellular staining for IFN-γ, TNFα and IL-2. The proportion of CD8+ T cells coproducing IFN-γ and TNFα (AC), or IFN-γ and IL-2 (BD) are shown for the influenza A viral epitopes DbNP366 (AB), and DbPA224 (CD). Data are representative of one experiment of 5 mice per group. Similar results were observed from two further recall experiments at day 55 after primary infection. (TIF) [file pone.0129768.s003.tif]

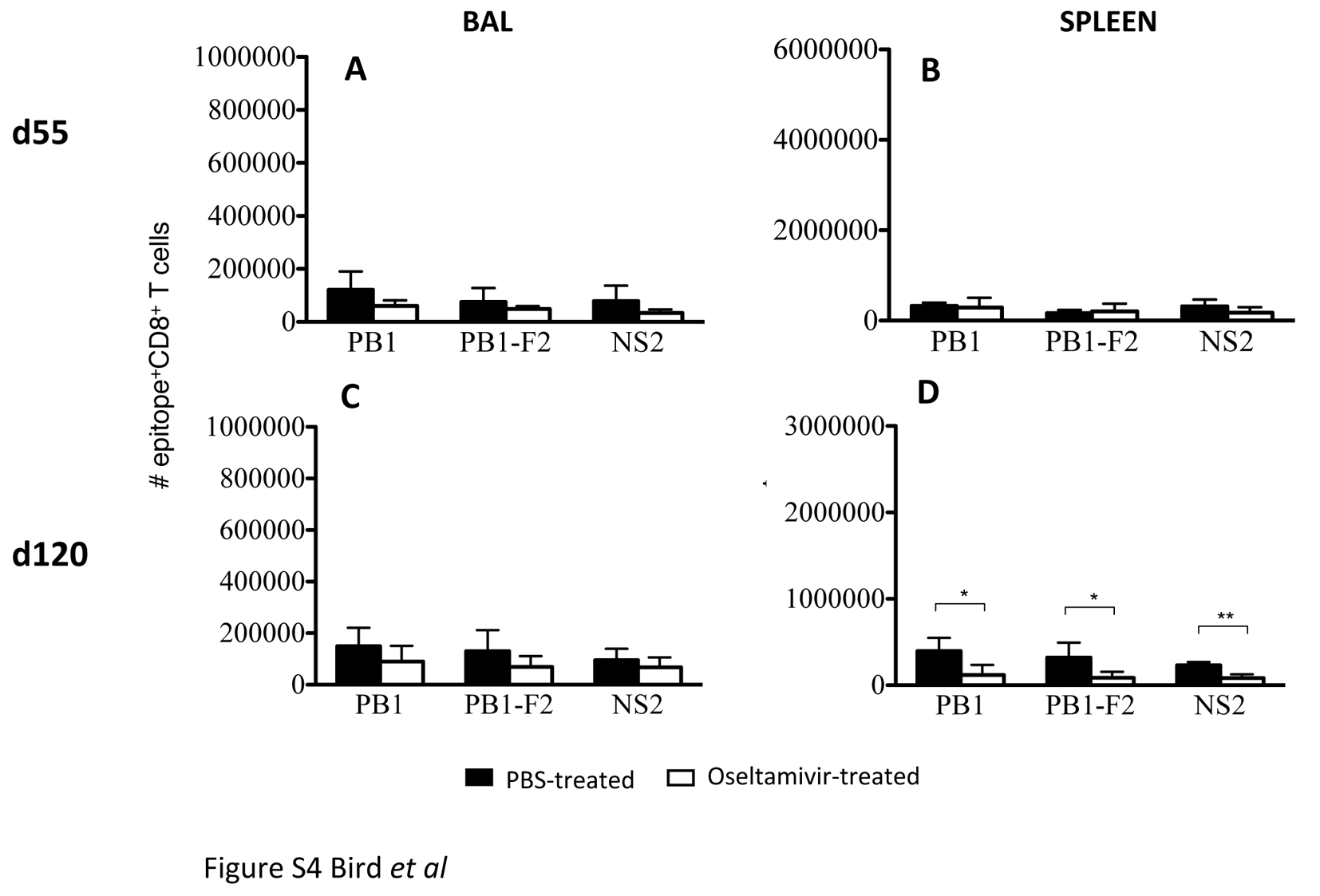

Supplement: S4 Fig — Naïve female BL/6 mice were administered either oseltamivir or PBS four hours prior to intranasal infection with 104 pfu of HK and then once daily for eight days. Mice were secondarily-challenged intranasally with 600 pfu of PR8 either (AB) 55, or (CD) 120 days after primary infection The less dominant KbPB1703, DbPB1-F262 and KbNS2114-specific CD8+ T cells in both BAL (AC) and spleen (BD) were enumerated by intracellular staining of IFN-γ after five hours of stimulation with cognate peptide. Data represent the mean and standard deviation of a single experiment with 4–5 mice per group and are plotted on the same scale as that of the immunodominant DbNP366 and DbPA224 (Fig 6) to demonstrate relative contributions to the secondary response. Similar results were observed from two additional recall experiments at day 55 after primary infection. (TIF) [file pone.0129768.s004.tif]
